# Awareness, Understanding, and Use of Nutrition Labels on Pre-Packaged Foods and Their Associations with Noncommunicable Diseases Among Adults in Shanghai, China

**DOI:** 10.3390/nu18050854

**Published:** 2026-03-06

**Authors:** Wei Zhou, Jingyi Si, Yifan Gao, Weiwei Zheng, Ruifen Li, Changfeng Zhu, Xue Han, Jiajie Zang, Zhengyuan Wang

**Affiliations:** 1 Department of School and Nutrition, Shanghai Yangpu District Center for Disease Control and Prevention (Shanghai Yangpu District Health Supervision Institute), Shanghai 200090, China; 2 Department of Gastroenterology and Hepatology, Zhongshan Hospital, Fudan University, Shanghai 200032, China; 3 General Office, Shanghai Yangpu District Center for Disease Control and Prevention (Shanghai Yangpu District Health Supervision Institute), Shanghai 200090, China; 4 Division of Health Risk Factors Monitoring and Control, Shanghai Municipal Center for Disease Control and Prevention, Shanghai 200336, China

**Keywords:** noncommunicable disease, nutrition label, generalized linear regression model, Shanghai

## Abstract

**Background**: Noncommunicable diseases (NCDs) are a major global public health challenge and can be prevented and managed through a balanced, nutrient-rich diet. Food nutrition labels play an important role in guiding healthier choices, particularly for individuals at risk of chronic health conditions. This study assessed awareness, understanding and use of nutrition labels among adults in Shanghai, China, and explored their associations with NCDs. **Methods**: A face-to-face structured questionnaire survey was conducted among 1503 adults in 2024. Data were collected on sociodemographic characteristics, self-reported chronic conditions (obesity, diabetes, hypertension, hyperlipidemia, hypercholesterolemia, cardiovascular and cerebrovascular diseases (CCVDs), and fatty liver disease), and awareness, understanding, and use of nutrition labels. Generalized linear regression models were applied to assess associations between label-related behaviors and chronic conditions. **Results**: Overall, 81.6% of participants were aware of labels, 15.0% reported understanding them, and 35.5% reported using them. Participants who were underweight or obese were less likely to be aware of labels compared to those with normal weight (73.8% and 72.9% vs. 83.5%). Individuals with fatty liver disease were less likely to understand labels compared to those without the condition (7.2% vs. 16.1%). Conversely, participants with three or more chronic conditions were more likely to use labels than those without any chronic conditions (46.1% vs. 34.4%). **Conclusions**: Among adults in Shanghai, nutrition label awareness was relatively high, while understanding and use of labels remained insufficient. Targeted nutrition education and the integration of nutrition labeling into chronic disease management strategies are needed to improve public health outcomes.

## 1. Introduction

Chronic noncommunicable diseases (NCDs) remain the leading cause of morbidity and mortality in China and globally [1,2]. In 2021, NCDs accounted for at least 43 million deaths, representing approximately 75% of non-pandemic-related fatalities worldwide, with 18 million individuals dying from an NCD before the age of 70 [3]. In China, NCD-attributed deaths increased from 80.0% in 2002 to 86.6% in 2012, and further to 88.5% in 2019 [4]. This prevalence is likely to increase over time due to factors such as population aging, urbanization, and lifestyle changes [5]. In Shanghai, among middle-aged and elderly populations, the prevalence of major NCDs ranked as follows: hypertension (55.3%), dyslipidemia (33.5%), diabetes (21.9%), obesity (12.4%), and osteoporosis (9.3%) [6].

Previous studies have reported that an unhealthy diet is one of the major preventable risk factors for a range of NCDs [7,8]. Nutrition interventions are essential in managing the risk of NCDs [8]. Some studies have shown that these interventions help populations to make healthier food choices, leading to improved health outcomes, including reduced energy intake, better glycemic control, and decreased cholesterol levels [9,10,11].

In China, efforts to promote healthy eating and combat nutrient-rerated NCDs have been made for several decades [12]. The Chinese government introduced a voluntary nutrition labelling code in 2007, which became mandatory on 1 January 2013. The standardized nutrition panel on pre-packaged foods includes mandatory declarations of energy, protein, fat, carbohydrates and sodium [13]. Nutrition labels provide consumers with information on the energy, fat, sodium and other nutrient content of food and beverages, thereby enhancing their understanding of the products they purchase and consume [14,15]. A 2016 study across 10 provinces in China reported that only 15.45% of urban residents generally or fully understand nutrition labels, while 36.41% reported often or always using them, a slightly lower rate was observed among rural residents [16]. Likewise, a study conducted in Shanghai between 2015 and 2016 found that only 19.3% of parents of primary and secondary school students used nutrition labels [17]. In-store observational research from the United Kingdom further suggests that actual label usage may be even lower, with only 27% of shoppers observed referring to nutrition information on packaging during supermarket visits [18].

To the best of our knowledge, although some studies have explored the knowledge, attitudes, and practices (KAP) regarding nutrition labels among the Chinese population [19,20,21,22], and research from countries such as the United States, Thailand and Mexico has reported varying associations between nutrition label awareness, use, and chronic diseases [23,24,25], few studies have systematically examined the awareness, understanding, and use of nutrition labels on pre-packaged foods and their associations with chronic health conditions—such as overweight, obesity, diabetes, hypertension, hyperlipidemia, hypercholesterolemia, cardiovascular and cerebrovascular diseases (CCVDs), and fatty liver disease—among Chinese adults. This study aims to address this gap by investigating the relationships between nutrition label awareness, understanding, and use, and these chronic conditions. The findings may provide valuable insights into how nutrition labels influence dietary behavior and health outcomes among adults in Shanghai.

## 2. Materials and Methods

### 2.1. Study Design and Participants

This cross-sectional study was conducted between April and June 2024 in five selected subdistricts of Yangpu District, Shanghai, China. The primary objective was to assess the awareness, understanding, and use of nutrition labels among adults and to examine their associations with chronic health conditions.

The survey targeted permanent residents of Yangpu District who had lived in the community for at least six months within the past 12 months and were aged 18 years or older. Convenience sampling was employed to recruit participants, and a pilot study was conducted with 150 participants. The anticipated level of understanding (*p*) of nutrient labelling among participants was 12%. The sample size calculation was as follows: *n* = (*u*^2^ × *p*(1 − *p*))/d^2^, where *u* = 1.96 at the 95% confidence level (two-sided), and *p* = 12%. The relative error (*r*) was set at 15%, and the allowable error (*d*) was calculated as *r* × *p*. The sample size was then increased by 10% to account for non-responses, resulting in a final sample size of 1392 residents. Exclusion criteria included individuals under 18 years old, those with diagnosed mental illness, illiteracy, members of the same household, or any condition that could hinder their compliance or cooperation with the survey.

This study was conducted in accordance with the principles of the Declaration of Helsinki. Ethical approval was obtained from the Ethics Committee of the Shanghai Yangpu District Center for Disease Control and Prevention (Shanghai Yangpu District Health Supervision Institute) (protocol code 2024/10, date of approval: 25 March 2024). Written informed consent was obtained from all participants.

### 2.2. Data Collection

Data were collected using a paper-based questionnaire designed for adults. Trained investigators conducted the survey within the five selected subdistricts. After the investigators explained the questionnaire completion procedure, participants were asked to complete the questionnaire anonymously and independently on site. Completed questionnaires were collected immediately to ensure data completeness and quality. The questionnaire focused on nutrition labels and chronic health conditions, and was adapted from previous studies conducted in China and Mexico on nutrition label knowledge, understanding, use, and self-reported chronic diseases [25,26]. Data were collected through face-to-face interviews conducted by trained and certified project staff. The questionnaire comprised three sections: (1) personal demographic information; (2) self-reported chronic health conditions; (3) awareness, understanding, and use of nutrient labels (Supplementary File S1). A multidisciplinary panel of experts in epidemiology, medicine, statistics, and nutrition reviewed and refined the questionnaire to ensure content validity, accuracy, and alignment with the study objectives. A total of 1550 questionnaires were distributed. Of these, 12 individuals declined to participate and 35 submitted incomplete responses. The final analysis included complete data from 1503 participants, resulting in a response rate of 96.97%.

### 2.3. Measures

#### 2.3.1. Demographic Factors

Individual demographic characteristics included gender (female; male), age (18–39 years, 40–59 years, and ≥60 years), education level (junior high school or below; high school or junior college; university or above), marital status (married; unmarried/divorced/widowed/others), and annual household income (low (≤CNY 149,999); mid-low (CNY 150,000–249,999); mid-high (CNY 250,000–349,999); high (≥CNY 350,000)). For reference, the average 2024 exchange rate was 1 USD ≈ 7.18 CNY. Participants also self-reported their height and weight which were used to calculate body mass index (BMI), defined as weight divided by height squared (kg/m^2^). BMI was classified into four categories according to Chinese classification criteria: underweight (BMI < 18.5 kg/m^2^), normal weight (18.5 kg/m^2^ ≤ BMI < 24 kg/m^2^), overweight (24 kg/m^2^ ≤ BMI < 28 kg/m^2^), and obesity (BMI ≥ 28 kg/m^2^) [27].

#### 2.3.2. Nutrition Label Questionnaire

The questionnaire included a series of items designed to assess participants’ awareness, understanding, and use of nutrition labels. The knowledge about labels comprised ten questions: (1) Are you aware of nutrition labels on pre-packaged foods? (2) How often do you observe nutrition labels when purchasing pre-packaged foods? (3) What information is mainly included on food nutrition labels? (4) Which indicators must be included on the nutrition labels of pre-packaged foods? (5) Which pre-packaged foods are required by national regulations to carry nutrition labels? (6) Which of the following is not a commonly used measurement unit on nutrition labels? (7) What does NRV (Nutrient Reference Values) represent on a nutrition label? (8) What information might be included on the nutrition facts label of “sugar-free crude-fiber biscuits”? (9) If the packaging of high-calcium milk is marked with “calcium helps strengthen bones and teeth”, what does it signify? (10) Among three food labels, which one indicates the healthiest option? Question 1 assessed awareness of nutrition labels, with a binary response (“yes” or “no”); participants answering “yes” were categorized as “aware”, and those answering “no” as “unaware”. Question 2 evaluated use of nutrition labels, with options: “always”, “often (6–9 times out of 10)”, “occasionally (3–5 times out of 10)”, “seldom (1–2 times out of 10)”, and “never”. Participants selecting “always” or “often” were categorized as “users,” while all other responses were classified as “non-users”. Understanding of nutrition labels was assessed using all ten questions. A “yes” response to Question 1 and “always” or “often” to Question 2 were considered correct. Each correct response to Questions 1–10 was awarded one point; incorrect or partially correct answers received zero. The sum of correct answers yielded a total understanding score ranging from 0 to 10. An ideal understanding of nutrition labels was defined as responding “yes” to Question 1 and achieving a total score of six or more.

#### 2.3.3. Self-Reported, Previously Diagnosed Chronic Diseases

The analysis focused on chronic diseases closely linked to poor or inadequate nutrition, including obesity, diabetes, hypertension, hyperlipidemia, hypercholesterolemia, CCVDs, fatty liver disease, and other related conditions. Except for obesity, which was assessed based on BMI, all health conditions were self-reported in our survey. Participants were identified as having a health condition if they answered “yes” to any of the following question regarding diagnoses made at a secondary or higher-level medical institution: “Have you ever been diagnosed with the following chronic diseases by a physician in a secondary or higher-level medical institution?” The listed conditions included: (1) diabetes, (2) hypertension, (3) hyperlipidemia, (4) hypercholesterolemia, (5) CCVDs, (6) fatty liver disease, (7) other related conditions, (8) none of the above.

### 2.4. Statistical Analyses

Descriptive statistical methods were used to analyze the sample. Frequencies and proportions (%) were calculated for categorical variables. Data normality distribution was tested by using the Kolmogorov–Smirnov test. Non-normally distributed variables are presented as the median and 25–75th percentiles (p25–p75) as appropriate. Generalized linear regression models, both unadjusted and adjusted for sociodemographic variables, were conducted to evaluate the associations between nutrition label awareness, understanding, and use and chronic conditions. For binary outcomes, a binomial distribution with a logit link was applied. In models where BMI categories (underweight, normal weight, overweight, and obesity) were included as the primary predictors, adjustments were made for gender, age, education level, marital status, and income. In models examining other chronic conditions (self-reported diabetes, hypertension, hyperlipidemia, hypercholesterolemia, CCVDs, fatty liver disease, and combinations of chronic conditions), BMI was additionally included as a covariate along with the aforementioned sociodemographic variables. All analyses were performed using Stata version 18.0 (StataCorp LP, College Station, TX, USA), and all figures were generated using GraphPad Prism version 10.0. A two-tailed *p*-value < 0.05 was considered statistically significant.

## 3. Results

### 3.1. Nutrition Label Awareness, Understanding and Use

The median (p25–p75) age of all respondents was 44 years (30–65 years), and 48.8% were male. The basic characteristics of study participants are shown in Table 1.

The survey found that 81.6% of participants were aware of nutrition labels. Those with higher education levels (high school or above) demonstrated greater awareness (84.3% for both high school or junior college and university or above) compared to those with junior high school education or below (70.0%) (high school or junior college: OR = 2.30; 95% CI, 1.66–3.19; *p* < 0.001; university or above: OR = 2.30; 95% CI, 1.63–3.26; *p* < 0.001). Additionally, individuals with higher annual household income exhibited greater awareness (89.8% for mid-high and 89.2% for high income) than those with low income (81.5%) (mid-high: OR = 2.01; 95% CI, 1.09–3.71; *p* = 0.025; high: OR = 1.88; 95% CI, 1.11–3.21; *p* = 0.020) (Table 1).

Of all participants, 15.0% demonstrated an ideal understanding of nutrition labels. The median (p25–p75) score of nutrition label knowledge was 4 (3–5), with a range of 0 and 9, 4.1% of participants scored 0. Additionally, more than one fifth of participants answered three or four questions correctly, accounting for 22.6% and 22.4%, respectively. Females demonstrated a high level of understanding (17.1%) compared to males (12.8%) (OR = 1.41, 95% CI, 1.06–1.87; *p* = 0.020). Participants aged 60 or older exhibited lower levels of understanding (11.2%) compared to those aged between 18–39 years old (17.1%) (OR = 0.61, 95% CI, 0.43–0.87; *p* = 0.006). Regarding education level, participants with high school education or above had a better understanding of nutrition labels (15.0% for high school or junior college, 18.5% for university or above) compared to those with junior high school education or below (8.6%) (high school or junior college: OR = 1.88, 95% CI, 1.18–3.00; *p* = 0.008; university or above: OR = 2.43; 95% CI, 1.51–3.89; *p* < 0.01) (Tabel 1).

Overall, 35.5% of respondents reported always or often using nutrition labels. Females reported more frequent use (40.9%) compared to males (29.7%) (OR = 1.64; 95% CI, 1.32–2.03; *p* < 0.001). Furthermore, individuals with mid-low annual household income reported lower use (30.3%) compared to those with low income (38.8%) (OR = 0.68; 95% CI, 0.54–0.87; *p* = 0.002) (Table 1).

### 3.2. Nutrition Label Awareness, Use, and Understanding in Relation to Self-Reported Chronic Conditions

Generalized linear regression models were used to explore the associations between nutrition label awareness, understanding, use, and chronic conditions. The model for BMI was adjusted for gender, age, education level, marital status and household income. Models for diabetes, hypertension, hyperlipidemia, hypercholesterolemia, CCVDs, fatty liver disease, and combinations of chronic conditions were additionally adjusted for BMI.

**Table 1:** Nutrition label awareness, understanding, and use by sociodemographic characteristics in Shanghai adults.

Variables	*n* (%)	Nutrition Label Awareness			Nutrition Label Understanding			Nutrition Label Use		
		*n* (%)	OR ^1^ (95% CI)	*p*-Value	*n* (%)	OR ^1^ (95% CI)	*p*-Value	*n* (%)	OR ^1^ (95% CI)	*p*-Value
Total	1503	1227 (81.6)			226 (15.0)			533 (35.5)		
Gender										
Male	733 (48.8)	589 (80.4)	Reference		94 (12.8)	Reference		218 (29.7)	Reference	
Female	770 (51.2)	638 (82.9)	1.18 (0.91, 1.53)	0.211	132 (17.1)	1.41 (1.06, 1.87)	0.020	315 (40.9)	1.64 (1.32, 2.03)	<0.001
Age group (years)										
18–39	638 (42.4)	522 (81.8)	Reference		109 (17.1)	Reference		216 (33.9)	Reference	
40–59	375 (25.0)	321 (85.6)	1.32 (0.93, 1.88)	0.121	62 (16.5)	0.96 (0.68, 1.35)	0.821	144 (38.4)	1.22 (0.93, 1.59)	0.145
≥60	490 (32.6)	384 (78.4)	0.81 (0.60 1.08)	0.149	55 (11.2)	0.61 (0.43, 0.87)	0.006	173 (35.3)	1.07 (0.83, 1.37)	0.612
Education level										
Junior high school or below	280 (18.6)	196 (70.0)	Reference		24 (8.6)	Reference		87 (31.1)	Reference	
High school or junior college	694 (46.2)	585 (84.3)	2.30 (1.66, 3.19)	<0.001	104 (15.0)	1.88 (1.18, 3.00)	0.008	255 (36.7)	1.29 (0.96, 1.73)	0.094
University or above	529 (35.2)	446 (84.3)	2.30 (1.63, 3.26)	<0.001	98 (18.5)	2.43 (1.51, 3.89)	<0.001	191 (36.1)	1.25 (0.92, 1.71)	0.152
Marital status										
Married	1046 (69.6)	860 (82.2)	Reference		149 (14.2)	Reference		384 (36.7)	Reference	
Unmarried/divorced/widowed/others	457 (30.4)	367 (80.3)	0.88 (0.67, 1.17)	0.379	77 (16.9)	1.22 (0.90, 1.65)	0.194	149 (32.6)	0.83 (0.66, 1.05)	0.126
Annual household income (CNY ^2^)										
Low (≤149,999)	577 (38.4)	470 (81.5)	Reference		79 (13.7)	Reference		224 (38.8)	Reference	
Mid-low (150,000–249,999)	631 (42.0)	493 (78.1)	0.81 (0.61, 1.08)	0.151	98 (15.5)	1.16 (0.84, 1.60)	0.367	191 (30.3)	0.68 (0.54, 0.87)	0.002
Mid-high (250,000–349,999)	128 (8.5)	115 (89.8)	2.01 (1.09, 3.71)	0.025	21 (16.4)	1.24 (0.73, 2.09)	0.426	44 (34.4)	0.83 (0.55, 1.23)	0.349
High (≥350,000)	167 (11.1)	149 (89.2)	1.88 (1.11, 3.21)	0.020	28 (16.8)	1.27 (0.79, 2.03)	0.319	74 (44.3)	1.25 (0.89, 1.78)	0.203

OR, odds ratio; CI, confidence interval; CNY, Chinese Yuan; ^1^ crude generalized linear models (each including only one predictor) were employed to assess variations in nutrition label awareness, understanding, and use across demographic variables; ^2^ values are presented in CNY. US dollar equivalents were calculated using an exchange rate of 1 USD ≈ 7.18 CNY (2024).

**Figure 1:**
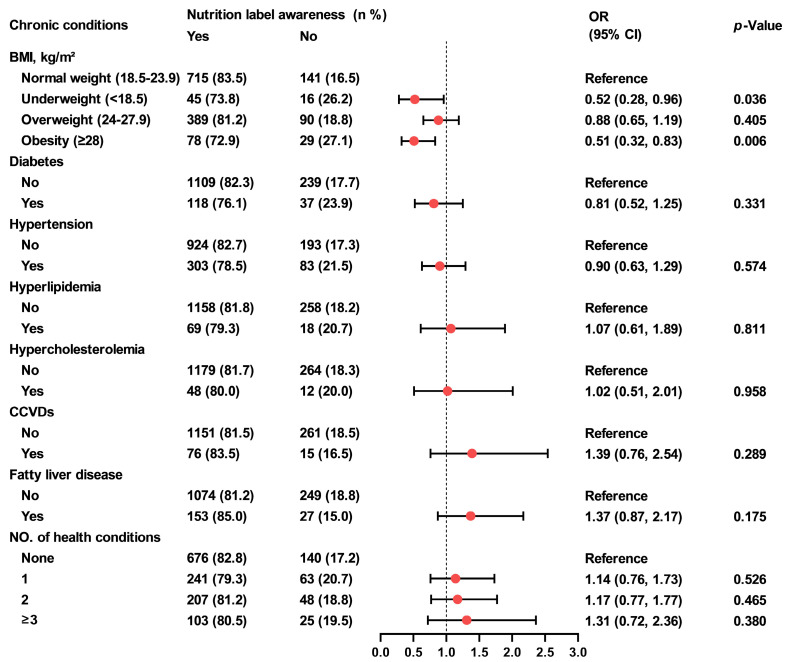
Association between food label awareness and chronic conditions among Shanghai adults. Generalized linear regression models were used to evaluate the associations between nutrition label awareness and chronic conditions. Generalized linear model (with predictors: overweight/obesity/underweight) was adjusted for gender, age, education level, marital status and income, generalized linear models (with predictors: self-reported diabetes, hypertension, hyperlipidemia, hypercholesterolemia, CCVDs, fatty liver diseases, and combinations of chronic conditions) were additionally adjusted for BMI. OR, odds ratio; CI, confidence interval; BMI, body mass index; CCVDs, cardiovascular and cerebrovascular diseases.

**Figure 2:**
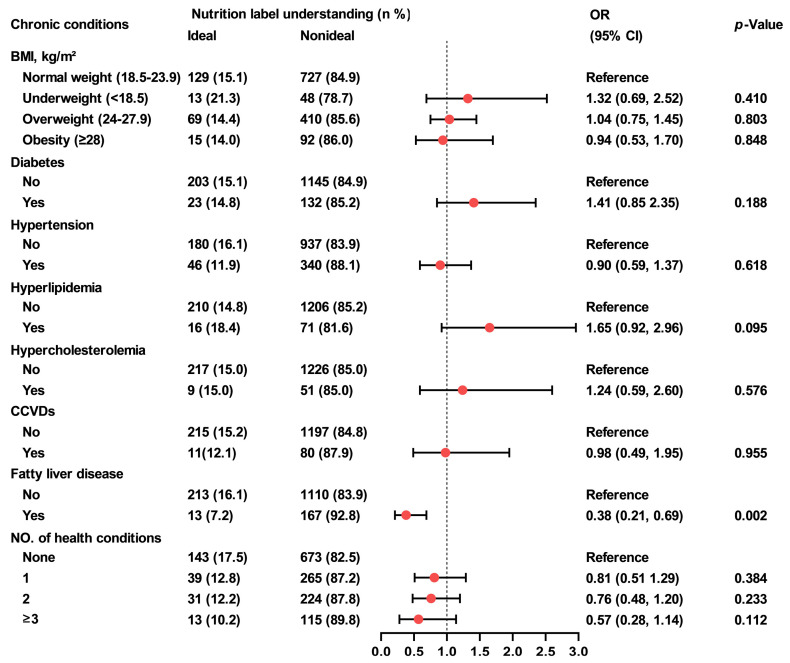
Association between food label understanding and chronic conditions among Shanghai adults. Generalized linear regression models were used to evaluate the associations between nutrition label understanding and chronic conditions. Generalized linear model (with predictors: overweight/obesity/underweight) was adjusted for gender, age, education level, marital status and income, generalized linear models (with predictors: self-reported diabetes, hypertension, hyperlipidemia, hypercholesterolemia, CCVDs, fatty liver diseases, and combinations of chronic conditions) were additionally adjusted for BMI. OR, odds ratio; CI, confidence interval; BMI, body mass index; CCVDs, cardiovascular and cerebrovascular diseases.

**Figure 3:**
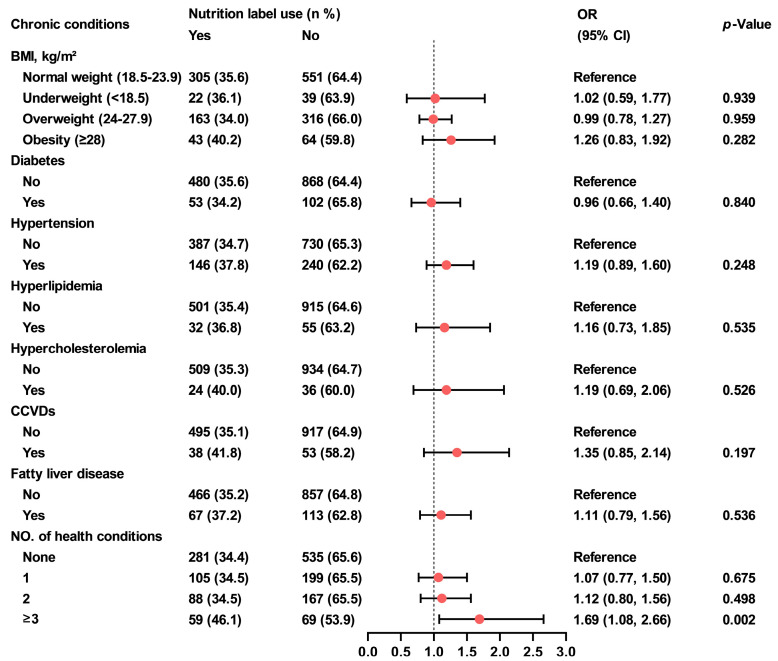
Association between food label use and chronic conditions among Shanghai adults. Generalized linear regression models were used to evaluate the associations between nutrition label use and chronic conditions. Generalized linear model (with predictors: overweight/obesity/underweight) was adjusted for gender, age, education level, marital status and income, generalized linear models (with predictors: self-reported diabetes, hypertension, hyperlipidemia, hypercholesterolemia, CCVDs, fatty liver diseases, and combinations of chronic conditions) were additionally adjusted for BMI. OR, odds ratio; CI, confidence interval; BMI, body mass index; CCVDs, cardiovascular and cerebrovascular diseases.

Participants who were underweight or obese showed lower awareness of nutrition labels compared to those with normal weight (73.8% and 72.9% vs. 83.5%; underweight: OR = 0.52, 95% CI, 0.28–0.96, *p* = 0.036; obesity: OR = 0.51, 95% CI, 0.32–0.83, *p* = 0.006) (Figure 1). Participants with fatty liver disease demonstrated poorer understanding of nutrition labels than those without the condition (7.2% vs. 16.1%; OR = 0.38, 95% CI, 0.21–0.69, *p* = 0.002) (Figure 2). Furthermore, individuals with three or more chronic conditions reported higher use of them than those without any chronic conditions (46.1% vs. 34.4%; OR = 1.69, 95%CI, 1.08–2.66, *p* = 0.002) (Figure 3).

## 4. Discussion

Since 2013, the Chinese government has mandated nutrition labels to improve consumer information regarding food purchases. This initiative has been promoted in China for nearly 12 years to educate people about healthy eating and to combat nutrient-related NCDs. This study assessed self-reported awareness, understanding, and use of nutrition labels among urban adults in Shanghai and examined their associations with sociodemographic characteristics and chronic conditions.

Our survey found that the majority (81.6%) of adults in Shanghai were aware of nutrition labels. However, a large percentage of the population reported low levels of understanding and use, only 15.0% demonstrated a good ability to understand them and just 35.5% often or always used them. A 2022 study of 1262 university students in Chongqing, China, revealed that only 21.3% understood nutrition labels on pre-packaged foods, while 48.4% of participants often or always used them [26], both of which was slightly higher than our findings. University students tend to have higher education levels and health awareness, which may explain these differences. A 2014 study by Jing et al. in Nanjing, China involving 427 consumers reported that over 70% of participants rarely or never used them [28], indicating a lower usage rate than observed in our study. This may be due to Shanghai’s relatively higher education levels, greater health awareness, and broader dissemination of nutrition information. A 2016 survey across 10 provinces in China indicated that only 15.45% of urban residents generally or fully understood nutrition labels, while 36.41% reported that they often or always used them [16]. These outcomes suggest that little or no progress has been made in this area over the past several years.

In this study, nutrition label awareness was associated with education and income; nutrition label understanding was influenced by gender, age, and education; while nutrition label use was determined by gender and income.

Gender influenced both the understanding and use of nutrition labels, with our study showing that females had a better understanding and were more likely to use them compared to males. However, gender did not significantly affect nutrition label awareness. Some previous studies also reported no significant gender differences in label awareness among college students and adults [29,30]. A five-country study conducted in 2018 across Australia, Canada, Mexico, the UK, and the USA also found that females demonstrated higher levels of understanding and use of nutrition facts tables [15,31], consistent with our findings.

Age had a negative impact on nutrition label understanding, suggesting that older individuals may have a poorer understanding of them. Participants aged 60 years or older demonstrated a significantly lower understanding compared to those aged 18–39. Similarly, a 2012 study by Liu et al. involving 660 consumers in China reported that age negatively affects both subjective and objective understanding [32], consistent with our findings. However, no significant differences in nutrition label awareness or use were observed across age groups in our study.

Education positively influenced both awareness and understanding of nutrition labels. In our study, individuals with a high school education or above demonstrated significantly greater awareness and better understanding compared to those with lower education levels. Income was positively associated with awareness but negatively associated with use. Participants with mid-high or high incomes demonstrated greater awareness than those with low incomes. However, individuals with mid-low incomes were less likely to use nutrition labels than those with low incomes. Our results align with those of Choi et al., who reported that lower awareness of food labelling was associated with lower education levels and household income [33]. Previous studies have also shown that lower levels of education and income are associated with reduced awareness, understanding, and use of nutrition labels, and less educated individuals tend to prefer simpler formats that highlight the overall healthiness of food products [21,34,35]. Our findings were partially consistent with these studies. Some studies have indicated that the use of nutrition labels on food choices was higher among individuals with high monthly incomes [36,37]. Speirs et al. reported that, among adults with low incomes, higher health literacy was associated with more frequent food label use, individuals with lower incomes generally exhibit lower nutritional literacy and lower rates of nutrition label use [38,39]. These findings are contrary to ours. One possible explanation is that individuals with mid-low incomes may have less spare time to shop or pay attention to the nutritional information on food products.

In our research, individuals who were underweight, obese or had fatty liver disease were less likely to be aware of or understand nutrition labels. A 2013 survey of 42,750 Thai adults found that participants who did not read, or understand nutrition labels, were more likely to report high blood pressure, high blood lipids, and obesity, but not an increased risk of being overweight [24]. These results are partially consistent with ours. However, Ibecheozor et al. reported that, among 3154 participants in the 2018 U.S. National Health and Nutrition Examination Survey (NHANES), among respondents with chronic illness, only obese patients were significantly more likely to notice caloric information compared to non-obese respondents [23]. This finding contrasts with ours and may be explained by differences in health awareness, medical guidance, and disease management strategies between populations in the two countries. Our results suggest that low awareness of nutrition labels may be associated with underweight and obesity, while poor understanding of nutrition labels may be associated with fatty liver disease. Further prospective cohort studies incorporating nutrition label awareness, understanding and chronic conditions are needed to test these hypotheses.

We also found that individuals with three or more chronic conditions were more likely to use nutrition label compared to those without any chronic conditions. However, previous studies on nutrition label use among patients with chronic disease have reported mixed findings. A Korean study based on 2008–2009 Korea NHANES data show that individuals diagnosed with hyperlipidemia, or those who had good control of it, were more likely to use them, whereas no such association was observed for hypertension or diabetes mellitus [40]. These findings are partially consistent with ours. Similarly, a U.S. study using 2005–2006 NHANES data found that individuals with chronic diseases generally reported better food label use compared to those without chronic diseases [41]. Another U.S. study using data from the 2007–2008 and 2009–2010 Flexible Consumer Behavior Survey found that frequent nutrition label use was associated with lower consumption of sodium and high-sodium foods [42]. One possible explanation is that individuals with chronic diseases may initially have less healthy lifestyles but become more health-conscious and modify their behaviors after diagnosis. However, a 2016 study by Nieto C et al. conducted in Mexico found that individuals with obesity, diabetes, or multiple chronic conditions were less likely to use nutrition labels than people without these conditions [25]. This finding contrasts with ours and may suggest that healthy individuals in Mexico may have healthier lifestyles and thus may have higher use of nutrition labels compared to those participants diagnosed with chronic conditions.

Our study has several notable strengths. To the best of our knowledge, it provides updated and comprehensive data on the awareness, understanding, and use of nutrition labels on pre-packaged foods in community-based populations in China, as well as their associations with chronic health conditions among adults. However, there are also some limitations. The findings were based on self-reported, previously diagnosed chronic diseases, which may be susceptible to recall bias. In addition, BMI was calculated based on self-reported height and weight, given individuals often underreport weight and overreport height, BMI may have been systematically underestimated, potentially introducing reporting bias and attenuating the observed associations. Second, participants were recruited using a convenience sampling approach within selected communities, which may limit the generalizability of the findings. The results should therefore be interpreted within the context of exploratory behavioral research. Moreover, the questionnaire was primarily designed as a structured assessment tool rather than a fully validated scale. Therefore, the instrument may not comprehensively capture the full complexity of related concepts, and the findings should be interpreted primarily in terms of awareness and understanding rather than deeper attitudinal measurement. Future studies should consider incorporating additional items and conducting more rigorous testing to further evaluate the reliability and validity of the instrument. Finally, as a cross-sectional study, causal relationships between nutrition label awareness, understanding, use, and chronic conditions cannot be established, and longitudinal or experimental studies are needed to clarify potential causal pathways.

## 5. Conclusions

In conclusion, despite widespread awareness of nutrition labels, the levels of understanding and use among adults in Shanghai remained low. Furthermore, individuals who were underweight, obese or had fatty liver disease showed lower levels of awareness or understanding, while those with three or more chronic conditions were more likely to use them than those without any chronic conditions. Our findings suggest that low awareness of nutrition labels may be associated with underweight and obesity, while poor understanding of nutrition labels may be associated with fatty liver disease. In contrast, a diagnosis of multiple chronic diseases may positively impact nutrition label use. Therefore, targeted nutrition education strategies, such as simplified nutrition label guidance, population-specific programs, and integration into chronic disease management should be developed to address the specific needs and barriers faced by different population groups in order to improve their nutrition label understanding and use.

## Data Availability

The original contributions presented in this study are included in the article. Further inquiries can be directed to the corresponding authors.

## Supplementary Materials

The following supporting information can be downloaded at: https://www.mdpi.com/article/10.3390/nu18050854/s1, Supplementary File S1: Nutrition Label Awareness, Understanding, and Use Questionnaire.

## Abbreviations

The following abbreviations are used in this manuscript:

NCDs	Noncommunicable diseases
CCVDs	Cardiovascular and cerebrovascular diseases
KAP	Knowledge, attitudes, and practices
CNY	Chinese Yuan
USD	United States Dollar
BMI	Body mass index
NRVs	Nutrient Reference Values
p25	25th percentiles
p75	75th percentiles
OR	Odds ratio
CI	Confidence interval
NHANES	National Health and Nutrition Examination Survey
