# Supplementary material for: Awareness, Understanding, and Use of Nutrition Labels on Pre-Packaged Foods and Their Associations with Noncommunicable Diseases Among Adults in Shanghai, China
Source: Nutrients. 2026 Mar 6;18(5):854. doi: 10.3390/nu18050854 (PMC12987300; doi:10.3390/nu18050854)
Supplement: Supplementary file 1 [file nutrients-18-00854-s001.zip › nutrients-4144457-supplementary.pdf]

Questionnaire Code □□□□□

Nutrition Label Awareness, Understanding, and Use Questionnaire

Dear Participant,

You are invited to participate in this survey, which aims to assess residents' awareness, understanding, and use of nutrition labels on prepackaged foods. Prepackaged foods refer to foods sold in stores or supermarkets with standardized packaging and labeling. The survey will take approximately 10 minutes to complete. All information collected will be kept confidential and used solely for research purposes. Your participation is voluntary, and you may withdraw at any time without consequence. Please answer the questions based on your actual situation.

Thank you for your participation.

Section 1. Demographic Characteristics

1. ID number

□□□□□□ □□□□ □□□□ □□□□

2. Residential address

Street______Road______Lane______No.______Room______

3. Survey date((YYYY-MM-DD)

□□□□ □□ □□

4. Gender

□ Male

□ Female

5. Age

□□ years old

6. Height

□□□.□ cm

7. Weight

□□□.□ kg

8. Contact number

□□□□□□□□□□□

9. Educational level

☐ No formal education

☐ Primary school

☐ Junior high school

☐ Senior high school or Technical secondary school

☐ Junior college or Vocational college

☐ Bachelor’s degree or above

10. Occupation

☐ Student

☐ Homemaker

☐ Unemployed

☐ Retired

☐ Managers of government agencies, party organizations, enterprises, and public institutions

☐ Professional and technical personnel

☐ Clerical and related staff

☐ Commercial and service personnel

☐ Agricultural, forestry, animal husbandry, fishery, and water conservancy workers

☐ Production and transportation equipment operators and related personnel

11. Marital status

☐ Single (never married)

☐ Married

☐ Widowed

☐ Divorced

☐ Others

12. Total household income in 2023

☐ Less than 29,000 RMB

☐ 29,000–49,999 RMB

☐ 50,000–99,999 RMB

☐ 100,000–149,999 RMB

☐ 150,000–199,999 RMB

☐ 200,000–249,999 RMB

☐ 250,000–349,999 RMB

☐ 350,000–399,999 RMB

☐ 400,000 RMB or above

Section 2. Health Status

13. Have you ever been diagnosed with any of the following chronic diseases by a physician in a secondary or higher-level medical institution?

☐ Diabetes

☐ Hypertension

☐ Hyperlipidemia

☐ Hypercholesterolemia

☐ Cardiovascular and cerebrovascular diseases

☐ Fatty liver

☐ Other chronic diseases:_______

☐ None of the above

Section 3. Awareness, Understanding, and Use of Nutrition Labels

14. Are you aware of nutrition labels on prepackaged foods?

☐ Yes

☐ No

15. How often do you observe nutrition labels when purchasing prepackaged foods?

☐ Every time

☐ Often (6-9 out of 10 times)

☐ Sometimes (3–5 out of 10 times)

☐ Rarely (1–2 out of 10 times)

☐ Never

16. What information is mainly included on food nutrition labels?

☐ Food name, ingredients, and instructions for use

☐ Production date, shelf life, and storage conditions

☐ Nutrition facts table, nutrition claims, and nutrient function claims

☐ Production batch number, manufacturer name, and address

☐ Don’t know

17. Which indicators must be included on the nutrition labels of prepackaged foods?

☐ Amino acids, protein, fat, vitamins, and minerals

☐ Energy, protein, fat, carbohydrates, and sodium

☐ Energy, amino acids, fat, carbohydrates, and food flavoring

☐ Water, starch, minerals, vitamins, monosodium glutamate, and spices

☐ Water, fat, starch, sodium, and food flavoring

18. Which prepackaged foods are required by national regulations to carry nutrition labels? (Multiple choice)

☐ Bottled purified water

☐ Sprite

☐ 40% alcohol Baijiu

☐ Lettuce

☐ Packaged sugar-free biscuits

☐ Instant noodles

☐ Fresh roasted chicken

☐ Vinegar

19. Which of the following is not a commonly used measurement unit on nutrition labels?

☐ Per 30 g

☐ Per 100 g

☐ 150g per bag

☐ Per 100 mL

☐ Per 500 g

20. What does NRV (Nutrient Reference Values) represent on a nutrition label?

Nutrition Facts Panel		
**Item**	Per 100g	NRV%
**Protein**	20g	33%

☐ The protein in 100 g of this food can meet 33% of the body’s daily requirement

☐ 33% of the protein in 100 g of this food can meet the daily requirement

☐ Protein makes up 33% of this 100 g food.

☐ 33% of the protein in 100 g of this food can be absorbed by the human body

☐ Don’t know

21. What information might be included on the nutrition facts label of “sugar-free crude-fiber biscuits”?

☐ Carbohydrates 56 g; Protein 0 g

☐ Carbohydrates 0 g; Protein 10 g

☐ Carbohydrates 56 g; Dietary fiber 10 g

☐ Carbohydrates 0 g; Dietary fiber 10 g

☐ Protein 0 g; Dietary fiber 0 g

22. If the packaging of high-calcium milk is marked with “calcium helps strengthen bones and teeth”, what does it signify?

☐ False advertising

☐ Inducing consumption

☐ Promoting nutritional functions

☐ Enhancing packaging appeal

☐ Don’t know

23. Among three food labels, which one indicates the healthiest option?

Image 1

Nutrition Facts Panel		
**Item**	Per 100g	NRV%
**Energy**	2209KJ	26%
**Fat**	4.8g	8%
--Trans fatty acids	4g	
**Calcium**	250mg	32%
**Vitamin D**	2.0μg	40%

Image 2

Nutrition Facts Panel		
**Item**	Per 100g	NRV%
**Energy**	1513KJ	18%
**Fat**	5.2g	9%
**Carbohydrate**	21g	0.07
**Vitamin C**	23μg	23%

Image 3

Nutrition Facts Panel		
**Item**	Per 100g	NRV%
**Energy**	1513KJ	16%
**Fat**	2.4g	4%
**Sodium**	1815mg	91%
**Dietary fiber**	1.8g	8%

☐ Image 1

☐ Image 2

☐ Image 3

☐ All of them

☐ Don’t know
